# First records of the genera *Sivaloka* Distant, 1906, with two new species from China, and description of a new species of genus *Kodaianella* Fennah, 1956 (Hemiptera, Fulgoromorpha, Issidae)

**DOI:** 10.3897/zookeys.917.47326

**Published:** 2020-03-09

**Authors:** Zhi-Min Chang, Lin Yang, Xiang-Sheng Chen

**Affiliations:** 1 Key Laboratory of Animal Genetics, Breeding and Reproduction in the Plateau Mountainous Region, Ministry of Education, College of Animal Science, Guizhou University, Guiyang, Guizhou, 550025, China Guizhou University Guiyang China; 2 Institute of Entomology/Special Key Laboratory for Developing and Utilizing of Insect Resources, Guizhou University, Guiyang, Guizhou, 550025, China Guizhou University Guiyang China; 3 The Provincial Key Laboratory for Agricultural Pest Management of Mountainous Regions, Guizhou University, Guiyang, Guizhou, 550025, China Guizhou University Guiyang China

**Keywords:** female genitalia, issid, Kodaianellini, new taxa, planthopper, *
Sivaloka
*

## Abstract

The genus *Sivaloka* Distant, 1906 (Hemisphaeriinae, Kodaianellini) is recorded from China for the first time, with two new species *Sivaloka arcuata* Chang & Chen, **sp. nov.** (China: Guizhou) and *Sivaloka trigona* Chang & Chen, **sp. nov.** (China: Guangxi). One new species of *Kodaianella* Fennah, 1956, *Kodaianella furcata* Chang & Chen, **sp. nov.** (China: Guangxi) is also described and illustrated; female genitalia of two known species in *Kodaianella* are described. A checklist of species of the tribe Kodaianellini with their distribution and a key to genera are provided.

## Introduction

The tribe Kodaianellini was established by Wang et al. (2016) for *Kodaianella* Fennah, 1956, which is the smallest tribe in the subfamily Hemisphaeriinae Melichar, 1906 (Hemiptera, Issidae), compared with Sarimini Wang, Zhang & Bourgoin, 2016, Parahiraciini Cheng & Yang, 1991, and Hemisphaeriini Melichar, 1906. The tribe Kodaianellini is characterized by hindwings with three lobes: Pcu-A_1_ lobe distinctly thinner, less than half as wide as the ScP-R-MP-Cu lobe; A_2_ lobe with anterior and posterior margins subparallel, and distinctly surpassing half-length of Pcu-A_1_ lobe; A_1_ branched, Pcu single and anastomosing with A_11_; A_2_ unbranched. Currently, the tribe Kodaianellini contains five genera: *Dentatissus* Chen, Zhang & Chang, 2014, *Kodaianella* Fennah, 1956, *Kodaianellissus* Wang, Bourgoin & Zhang, 2017, *Neokodaiana* Yang, 1994, and *Tetricissus* Wang, Bourgoin & Zhang, 2017 (Distant 1906; Fennah 1956; Chan and Yang 1994; Chen et al. 2014; Wang et al. 2017), and we here add *Sivaloka* Distant, 1906, which is transferred from the tribe Issini Spinola, 1839. These six genera with all 11 species mainly distributed in the Oriental region, exceptionally a few present in the Palaearctic region (Bourgoin 2019).

In China, the type genus *Kodaianella* was fixed by Fennah (1956) with *K. bicinctifrons*, from Sichuan Province in Southwest China, as its type. Chou et al. (1985) mistakenly recorded a second genus *Sivaloka* Distant, 1906 and described a new species, *S. damnosus* Chou & Lu, 1985, a species which causes serious damage to apple trees. Zhang and Chen (2010) reviewed the genus *Kodaianella* and added two more species, *K. longispina* Zhang & Chen, 2010 and *K. machete* Zhang & Chen, 2010. However, Gnezdilov (2013) placed *K. machete* Zhang & Chen, 2010 in synonymy with *Sivaloka damnosa* Chou & Lu, 1985, and transferred *S. damnosa* and *S. bipartita* Distant, 1906 to *Kodaianella*. Chen et al. (2014) transferred *Kodaianella damnosa* into a new genus *Dentatissus*, and added one more species. At present, with the exception of genera *Sivaloka* and *Tetricissus*, the other four genera with seven species are recorded in the tribe Kodaianellini from China.

We record the genus *Sivaloka* in China for the first time and describe two new species from Guizhou and Guangxi. An additional new species of *Kodaianella* from Guangxi is also described and illustrated, and the female genitalia of two known species are described. A checklist of all species of the tribe Kodaianellini, with their distribution, and a key to genera are provided.

## Materials and methods

The morphological terminology of the head and body follows Chan and Yang (1994) and Bourgoin et al. (2015) for the wing venation, and Bourgoin (1987, 1993) and Gnezdilov (2002, 2003) for male and female genitalia. Dry specimens were used for descriptions and illustrations. External morphology was observed under a stereoscopic microscope. All measurements are in millimeters (mm). The body measurements are from the apex of vertex to the tip of the forewings. The genital segments of the examined specimens were macerated in 10% NaOH, washed in water, and transferred to glycerin. Illustrations of the specimens were made with a Leica M125 and Olympus CX41 stereomicroscope. Photographs were taken with Keyence VHX-1000C and Nikon SMZ-25 microscopes.

The type specimens and other examined specimens are all deposited in the Institute of Entomology, Guizhou University, Guiyang, China (**IEGU**).

### Checklist of genera and species of Kodaianellini Wang, Zhang & Bourgoin, 2016 of the world

*Dentatissus* Chen, Zhang & Chang, 2014

*Dentatissus brachys* Chen, Zhang & Chang, 2014: China (Henan).

*Dentatissus damnosus* (Chou & Lu, 1985): China (Beijing, Guizhou, Henan, Hubei, Jiangsu, Liaoning, Shaanxi, Shangdong, Shanxi, Sichuan, Yunnan, Zhejiang).

*Kodaianella* Fennah, 1956

*Kodaianella bicinctifrons* Fennah, 1956: China (Guizhou, Sichuan), Laos.

*Kodaianella bipartita* (Distant, 1906): Myanmar.

*Kodaianella furcata* Chang & Chen, sp. nov.: China (Guangxi).

*Kodaianella longispina* Zhang & Chen, 2010: China (Yunnan).

*Kodaianellissus* Wang, Bourgoin & Zhang, 2017

*Kodaianellissus intorqueus* Wang, Bourgoin & Zhang, 2017: China (Yunnan).

*Neokodaiana* Yang, 1994

*Neokodaiana chihpenensis* Yang, 1994: China (Taiwan).

*Neokodaiana minensis* Meng & Qin, 2016: China (Fujian).

*Neokodaiana yaeyamana* Gnezdilov & Hayashi, 2015: Nansei-shoto (Ryukyu Islands).

*Sivaloka* Distant, 1906

*Sivaloka arcuata* Chang & Chen, sp. nov.: China (Guizhou).

*Sivaloka limacodes* Distant, 1906: India.

*Sivaloka trigona* Chang & Chen, sp. nov.: China (Guangxi).

*Tetricissus* Wang, Bourgoin & Zhang, 2017

*Tetricissus philo* (Fennah, 1978): Vietnam.

### Key to genera of Kodaianellini Wang, Zhang & Bourgoin, 2016 of the world

**Table:** 

1	Forewings with Pcu+A_1_ veins keel-shaped (Figs 29, 44)	*** Sivaloka ***
–	Forewings with Pcu+A_1_ veins non keel-shaped	**2**
2	Hindwings with A_11_ vein branched	**3**
–	Hindwings with A_11_ vein simple, unbranched	**4**
3	Hindwings with A_2_ lobe distinctly narrower than Pcu-A_1_ lobe, and A_2_ vein nearly reaching to middle of A_2_ lobe (Wang et al. 2017: fig. 6)	*** Kodaianellissus ***
–	Hindwings with A_2_ lobe as wide as Pcu-A_1_ lobe, and A_2_ vein nearly reaching to apical margin of A_2_ lobe (Wang et al. 2017: fig. 23)	*** Tetricissus ***
4	Frons with one pale transverse carina in middle level of frons, and one pale transverse band above frontoclypeal suture (Gnezdilov and Hayashi 2015: figs 1, 2)	*** Neokodaiana ***
–	Frons without above characters	**5**
5	Anal tube with the maximum width near apical margin in dorsal view (Fig. 9); aedeagus with one hooked process in lateral view (Fig. 12)	*** Kodaianella ***
–	Anal tube with the maximum width near middle in dorsal view (Chen et al. 2014: fig. 2–79H); aedeagus with two hooked processes in lateral view (Chen et al. 2014: fig. 2–79K)	*** Dentatissus ***

## Taxonomy

**Family Issidae Spinola, 1839**

**Subfamily Hemisphaeriinae Melichar, 1906**

**Tribe Kodaianellini Wang, Zhang & Bourgoin, 2016**

### Genus Kodaianella

Taxon classificationAnimaliaHemipteraIssidae

Fennah, 1956

DAA93313-15B5-5F3C-90E6-F07518FA770A

Kodaianella Fennah 1956: 508; Zhang and Chen 2010: 62; Gnezdilov 2013: 42; Chen et al. 2014: 136.

#### Type species.

*Kodaianella bicinctifrons* Fennah, 1956.

#### Diagnostic characters.

**Figures 1–13.**
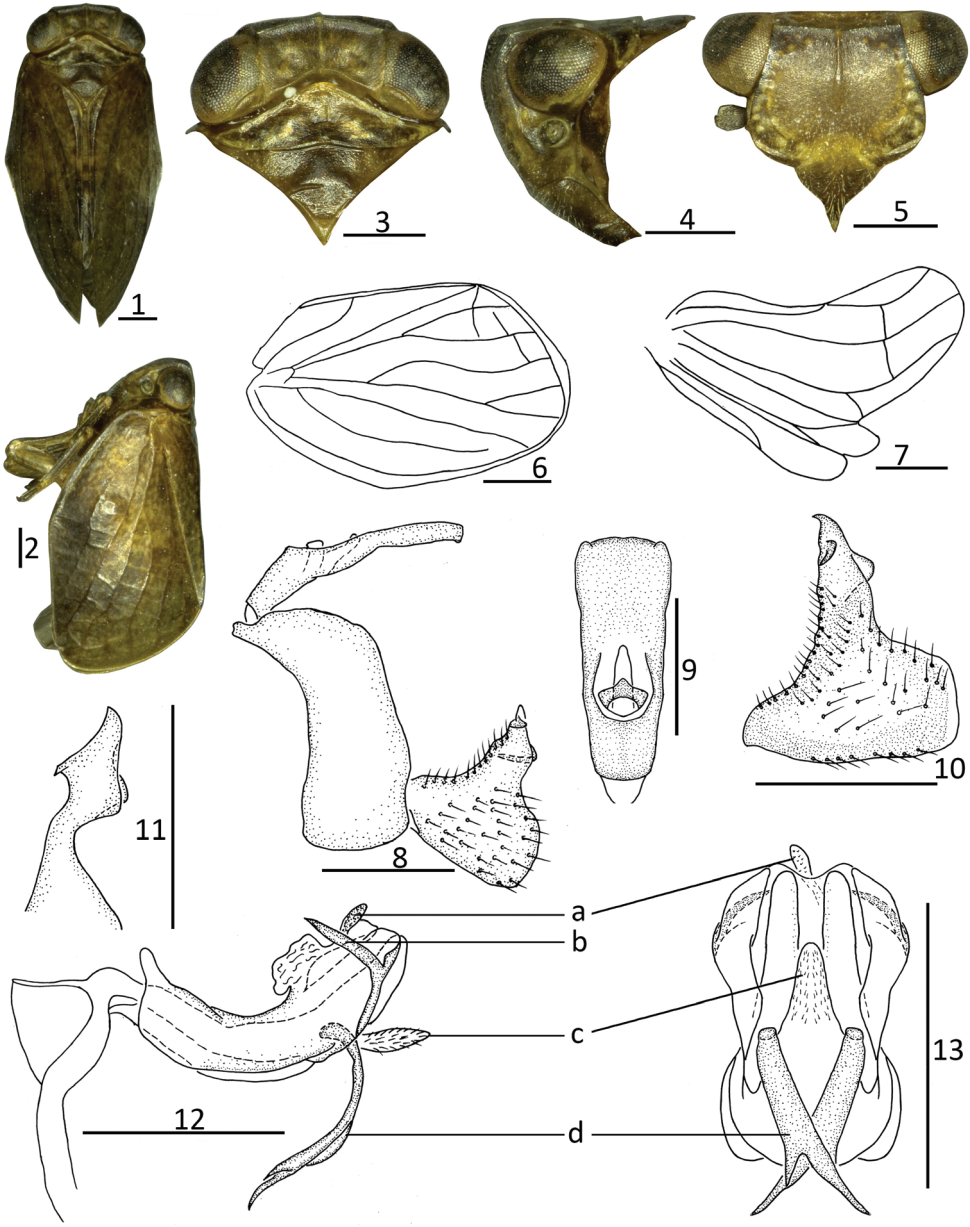
*Kodaianella furcata* Chang & Chen, sp. nov. **1** habitus, dorsal view **2** habitus, lateral view **3** head and thorax, dorsal view **4** head and thorax, lateral view **5** head, ventral view **6** forewing **7** hindwing **8** male genitalia, lateral view **9** male anal segment, dorsal view **10** genital styles, lateral view **11** capitulum of genital styles, ventral view **12** phallobase and aedeagus, lateral view **13** phallobase and aedeagus, ventral view. Scale bars: 0.5 mm. Abbreviations: a = rod-like process; b = hook-like process; c = ventral lobe; d = long forked process.

Body size small, slightly flat in dorsal view (Fig. 1). Width of head (Figs 1, 3) including eyes, narrower than pronotum. Vertex (Fig. 3) irregularly quadrangular, with width at base ca 1.7–2.3 times longer than length in middle; disc of vertex slightly depressed, with median carina linear or obscure; anterior margin slightly arched, convex; posterior margin obviously arched or obtusely concave. Gena in lateral view (Fig. 4) with one obvious ocellus between compound eye and antenna. Frons (Fig. 5) irregularly hexagonal, nearly flat, with median carina explicit and straight, reaching to 2/3 of frons, without lateral carinae; maximum width broader than length in middle; base slightly narrow, broader toward to apical margin; lateral margins of frons incurved below level of socket of antennae, with verrucae near lateral margins. Clypeus (Fig. 5) triangular, with median carina obscure or absent. Rostrum (Fig. 5) nearly surpassing mesotrochanters. Pronotum (Figs 1, 3) triangular, with median carina distinct or obscure and degraded, with distinct lateral carinae, without sub-lateral carinae; apical margin obtusely angled convex; posterior margin slightly arched or nearly straight. Mesonotum (Fig. 3) triangular, with median carina obvious or obscure or absent, sub-lateral carinae absent. Forewings (Figs 1, 2, 6) irregularly quadrangular, length ca 1.6–2.4 times longer than maximum width; anterior margin obviously arched; posterior margin straight; apical margin nearly truncated; longitudinal veins obvious and short transverse veins numerous and not obvious; with “hypocostal plate”, ScP and RP convergent near base, ScP and RP veins long, not forked, nearly reaching apical margin of forewings; MP bifurcating into two branches near base; CuA forked into two branches near middle; CuP present; Pcu and A_1_ united near middle of clavus. Hindwings (Fig. 7) with three lobes: ScP-R-MP-Cu lobe developed; Pcu-A_1_ lobe distinctly thinner, less than half as wide as ScP-R-MP-Cu lobe; A_2_ lobe thinner, distinctly surpassing half-length of Pcu-A_1_ lobe, anterior and posterior margins subparallel; Pcu simple, anastomosing with A_11_; A_11_ unbranched, A_12_ simple and straight; A_2_ unbranched, not reaching to apical margin of hindwings. Hind tibiae with 2 lateral spines in distal half and 8–10 apical spines; first metatarsomere with 7–11 apical spines; second metatarsomere with 2 apical spines; spinal formula of hind leg (2)8–10/7–11/2.

#### Male genitalia.

Anal tube (Figs 8, 9) irregularly triangular, longer in middle than maximum width in dorsal view, basal part narrow, apical part broader, maximum width near apical margin. Anal style (Fig. 9) moderately long, not surpassing anal tube. Pygofer (Fig. 8) symmetrical, irregularly rectangular; anterior and posterior margins nearly parallel in lateral view; dorsal and ventral margins nearly parallel in lateral view. Genital styles (Figs 8, 10) irregularly triangular; dorso-anterior margin not obvious, dorso-posterior margin and ventral margin nearly parallel. Capitulum of genital styles obvious and long (Fig. 11). Phallobase (Figs 12, 13) symmetrical, “U”-liked tubular in lateral view; dorsal lobe with apical part membranous, with various processes in lateral view. Aedeagus (Figs 12, 13) with one hooked process in lateral view.

#### Female genitalia

**Figures 14–22.**
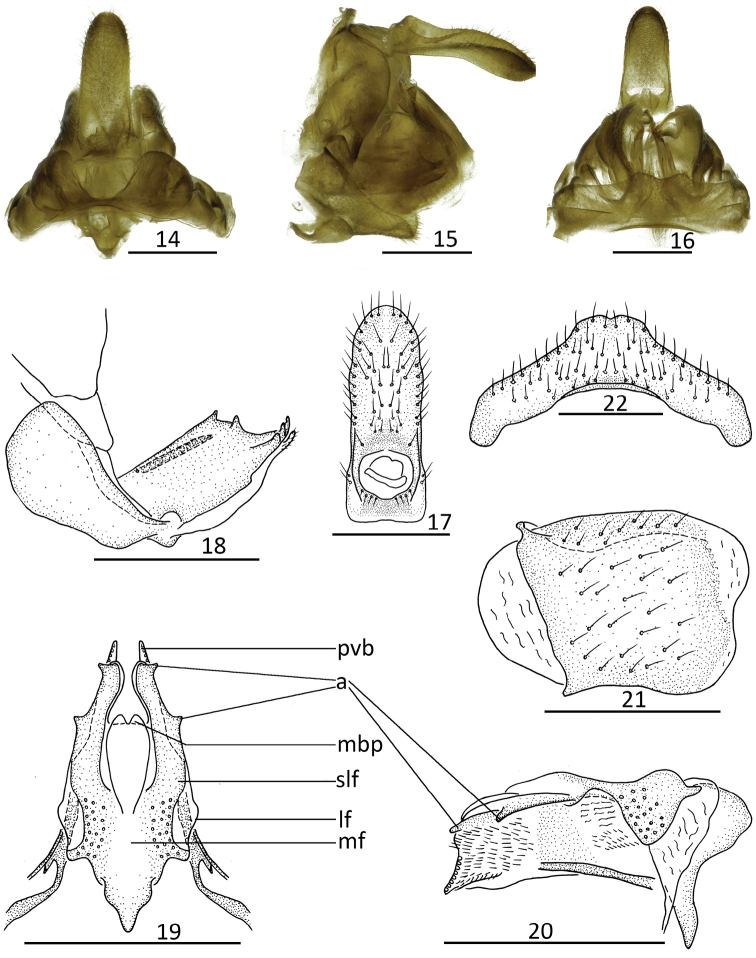
Female genitalia. *Kodaianella bicinctifrons* Fennah, 1956 **14** overall, dorsal view **15** overall, lateral view **16** overall, ventral view **17** female anal segment, dorsal view **18** anterior connective lamina of gonapophyses VIII, lateral view **19** posterior connective lamina of gonapophyses IX, dorsal view **20** posterior connective lamina of gonapophyses IX, lateral view **21** gonoplacs, lateral view **22** sternite VII, ventral view. Scale bars: 0.5 mm. Abbreviations: lf = lateral field of posterior connective lamina of gonapophyses IX; slf = sublateral field of posterior connective lamina of gonapophyses IX; mf = medial field of posterior connective lamina of gonapophyses IX; mdp = medial dorsal process; pvb = posterior ventral lobes; a = triangular process.

**Figures 23–28.**
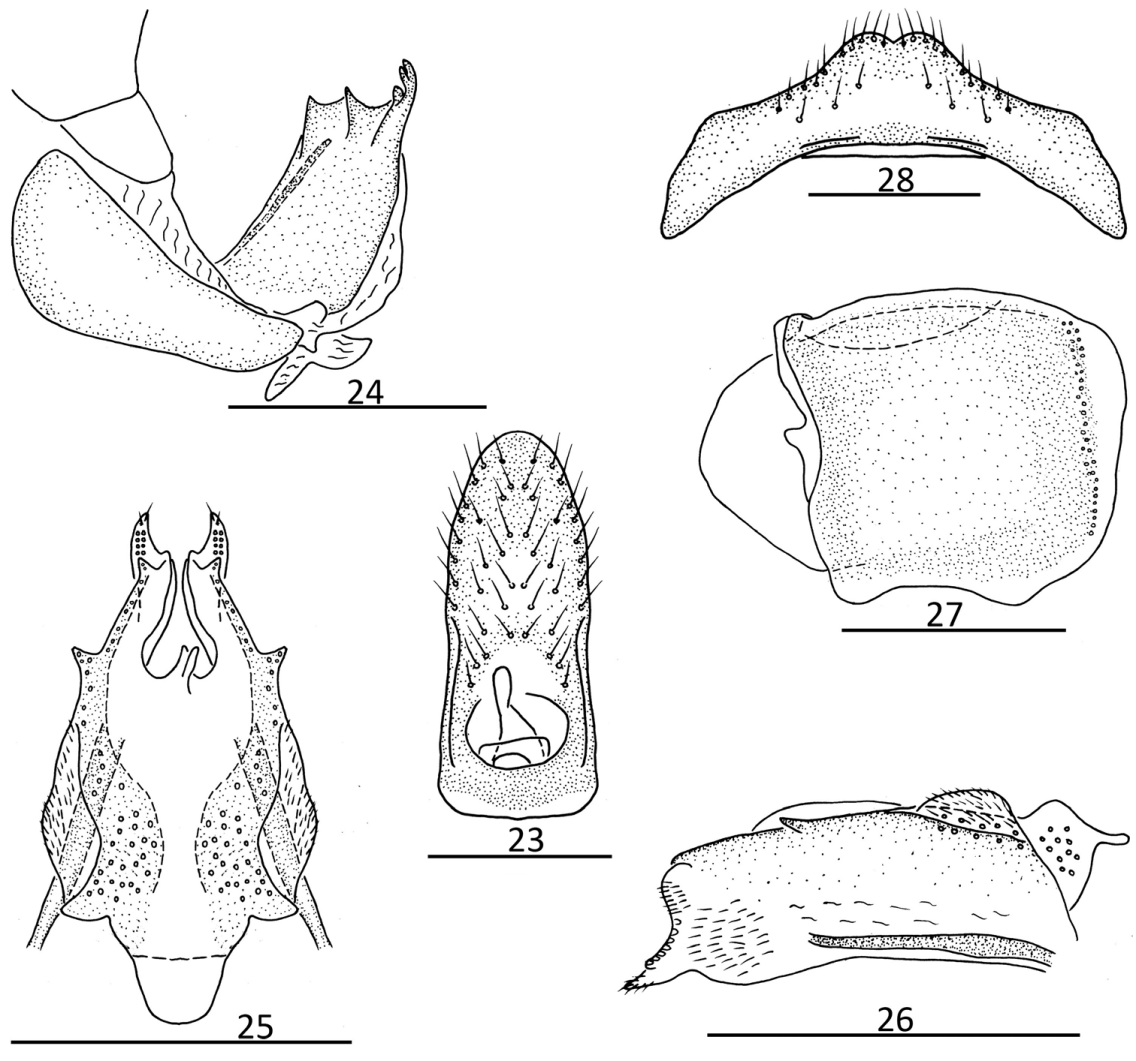
Female genitalia. *Kodaianella longispina* Zhang & Chen, 2010 **23** female anal segment, dorsal view **24** anterior connective lamina of gonapophyses VIII, lateral view **25** posterior connective lamina of gonapophyses IX, dorsal view **26** posterior connective lamina of gonapophyses IX, lateral view **27** gonoplacs, lateral view **28** sternite VII, ventral view. Scale bars: 0.5 mm.

(Figs 14–28). Anal tube (Figs 17, 23) oblong, obviously longer in middle than its width; apical margin arched, convex; lateral margin parallel. Anal style (Figs 17, 23) long or short, located near base of anal tube. Hind margin of gonocoxa VIII with endogonocoxal lobe not obvious (Figs 18, 24); endogonocoxal process membranous, gradually narrowing. Anterior connective lamina of gonapophyses VIII (Figs 18, 24) irregularly rectangular, with two lateral teeth bearing two keels in lateral group and three teeth in apical group. Posterior connective lamina of gonapophyses IX (Figs 19, 20, 25, 26) triangular, with lateral field membranous; sublateral field with two sclerous triangular processes in lateral margin (Figs 19, 25); median field with unobvious prominence (median dorsal process) (Figs 19, 25); ventroposterior lobes acutely bent at an angle (posterior ventral lobes) (Figs 20, 26). Gonoplacs (Figs 21, 27) irregularly rounded, without keels. Hind margin of sternite VII (Figs 22, 28) median area raised in ventral view, with small incision in middle.

### Key to species of *Kodaianella* Fennah, 1956 of the world

**Table:** 

1	First metatarsomere of hind legs with 7 apical teeth (Gnezdilov 2013: fig. 4)	***K. bipartita***
–	First metatarsomere of hind legs with more than 7 apical teeth	**2**
2	Anal tube with apical margin nearly truncated near middle part (Fig. 9); aedeagus with pair of biforked long hooks in ventral view (Fig. 13)	***K. furcata* Chang & Chen, sp. nov.**
–	Anal tube with apical margin concave near middle; aedeagus with pair of simple long hooks in ventral view	**3**
3	Phallobase with pair of long spines near apical part (Zhang and Chen 2010: fig. 25)	***K. longispina***
–	Phallobase with pair of short spines near apical part (Zhang and Chen 2010: fig. 7)	***K. bicinctifrons***

### Kodaianella furcata

Taxon classificationAnimaliaHemipteraIssidae

Chang & Chen sp. nov.

B32F475B-7714-5B73-A804-0D59020FC0C4

http://zoobank.org/EB9B5313-A0A2-4F2B-821F-FD5E8B5A342E

Figs 1–13

#### Type material.

Holotype: ♂, China: Guangxi, Nonggang National Nature Reserve (22°28'N, 106°58'E), 8 May 2011, H Li leg.; paratypes: 5♂♂, same data as holotype (IEGU); 1♂, Guangxi, Nonggang National Nature Reserve (22°28'N, 106°58'E), 7–8 May 2012, H Li and N-N Yang, leg.

#### Diagnosis.

This species is similar to *K. longispina* Zhang & Chen, 2010 in appearance, but it differs from the latter in having the phallobase with dorsal lobe bearing one rod-like process near its apical part in lateral view (Fig. 12a); the phallobase with ventral lobe distinct short, apical part finger-liked in ventral view (Fig. 13c); and the aedeagus in lateral view, with a forked and hooked process near the apical 1/3 (Fig. 12d).

#### Description.

Body length: male 3.94–4.18 mm; forewing: male 3.16–3.45 mm.

#### Coloration.

General color brown (Figs 1, 2). Vertex, pronotum, and mesonotum (Fig. 3) black-brown. Frons (Fig. 5) black-brown, with pale yellow verrucae along base and lateral margins. Clypeus (Fig. 5) yellow-brown. Compound eyes black-brown, ocelli pale green (Fig. 4). Forewings (Fig. 2) yellow-brown, with dark spots. Legs yellow-brown, with tips of spines on hind tibiae and tarsi black.

#### Head and thorax.

Head (Fig. 3) including eyes, slightly narrower than pronotum (0.96: 1.00). Vertex (Fig. 3) shorter in middle than the width at base (0.43: 1.00), with median carina linear; anterior margin slightly convexly arched; posterior margin obviously obtusely concave. Frons (Fig. 5) shorter in middle than the widest breadth (0.60: 1.00); median carina obvious and straight, reaching to 2/3 level of frons. Clypeus (Fig. 5) triangular, without median carina. Pronotum (Fig. 3) with median carina obscure, lateral carina reaching to the posterior margin. Mesonotum (Fig. 3) with median carina obscure. Forewings (Fig. 6) 1.60 times as long as maximum breadth; with wide “hypocostal plate”; ScP and RP convergent near base, ScP and RP veins long, nearly reaching apical margin; MP bifurcating two branches near basal 1/3, MP_1_ forked near apical 1/3; CuA forked into two branches near middle; CuP present, Pcu and A_1_ united near middle of clavus. Hindwings (Fig. 7) with ScP+R and M simple, not forked, CuA forked near apical part, with one vein between R and M, M and CuA_1_; and Pcu and A_11_ jointed near apical 1/4, without short transverse vein between Pcu + A_11_ and A_12_; A_2_ simple, reaching to 2/3 of A_2_ lobe. Spinal formula of hind leg (2)8/8/2.

#### Male genitalia.

Anal tube (Fig. 9) longer in midline than the width (2.49: 1.00) in dorsal view; lateral margins nearly parallel and widest in apical part; apical margin nearly truncated, with unobvious small, angular process near lateral margin. Anal style (Fig. 9) stout and long, located at the base 2/5 of anal tube, surpassing the opening of anal pore. Pygofer (Fig. 8) irregularly rectangular; dorsal margin slightly broader than ventral margin; anterior margin arched near dorsal 1/3; posterior margin nearly straight. Genital styles (Figs 8, 10) relatively triangular; anterior margin without triangular process; posterior margin with triangular process. Capitulum of genital styles irregularly triangular, with small irregular triangular, relatively long and stout neck (Fig. 11). Phallobase (Figs 12, 13) with dorsal lobe cystiform near apical part, with stout rod-like process (Figs 12a, 13a) in apical 1/6, directed to posterior, with dorso-lateral lobe with short hook-like process (Fig. 12b), pointed to dorsal margin in lateral view; lateral lobe splitting into two stout branches; ventral lobe membranous, apical part narrow, surface with microvilli in lateral view (Fig. 12c); ventral lobe in ventral view obviously shorter than dorsal lobe, with apical part projecting into finger-like process in middle (Fig. 13c). Aedeagus (Figs 12, 13) with long, hooked process near apical 1/3 in ventral view, tip of process directed to ventro-posterior in lateral view (Fig. 12d); in ventral view, hooked process forked into asymmetrical hooks (Fig. 13d).

#### Etymology.

The new species is derived from the Latin word “*furcata*”, in reference to the aedeagus, which bears a forked, hooked process.

#### Host plant.

Unknown.

#### Distribution.

China (Guangxi).

### Kodaianella bicinctifrons

Taxon classificationAnimaliaHemipteraIssidae

Fennah, 1956

9D87BC0F-C681-5732-9A33-D417786B01AF

Figs 14–22

Kodaianella bicinctifrons Fennah 1956: 508; Chen et al. 2014: 137.

#### Material examined.

1♂2♀♀, China: Guizhou, Congjiang County, 24 June 2005, D-Y Ge leg.; 2♂♂1♀, Sichuan, Kangding County, 8 Aug. 2005, F-L Xu leg.

#### Female genitalia

(Figs 14–16). Anal tube (Fig. 17) longer in middle than its width (2.64: 1.00). Anal style (Fig. 17) short, located in basal 1/4 of anal tube, not surpassing the opening of anal pore. Hind margin of gonocoxa VIII with endogonocoxal lobe not obvious, endogonocoxal process membranous, gradually narrowing (Fig. 18). Anterior connective lamina of gonapophyses VIII (Fig. 18) irregularly rectangular; with two lateral teeth bearing two keels in lateral group and three teeth in apical group. Posterior connective lamina of gonapophyses IX (Figs 19, 20) triangular, narrow, with lateral field membranous; sublateral field sclerous, with one triangular process in outer lateral margin near middle and another triangular process in apical part (Figs 19a, 20a); median field with symmetrical mountain-like prominence, apical margin concave (median dorsal process); ventroposterior lobes acutely bent at an angle (posterior ventral lobes). Gonoplacs (Fig. 21) without keels. Hind margin of sternite VII (Fig. 22) median area raised in ventral view, with shallow incision.

### Kodaianella longispina

Taxon classificationAnimaliaHemipteraIssidae

Zhang & Chen, 2010

00762661-091A-5BC5-8803-425070D9530B

Figs 23–28

Kodaianella longispina Zhang & Chen 2010: 66; Chen et al. 2014: 140.

#### Material examined.

2♂♂3♀♀ (paratypes), China: Yunnan, Baoshan, 8–20 Aug. 2006, P Zhang, Z-G Zhang and Q-Z Song, leg.

#### Female genitalia.

As in *K. bicinctifrons* Fennah, 1956, but anal tube longer in middle than the width (2.18: 1.00); anal style long, surpassing the opening of anal pore (Fig. 23). Anterior connective lamina of gonapophyses VIII (Fig. 24) with two lateral teeth bearing two keels in lateral group and three teeth in apical group. Posterior connective lamina of gonapophyses IX (Fig. 25) broader, with median field with irregular, thin prominence; distal part of ventroposterior lobes bent at an obvious angle (Fig. 26). Gonoplacs (Fig. 27) without keels. Hind margin of sternite VII (Fig. 28) median area raised in ventral view, with deeper incision.

### Genus Sivaloka

Taxon classificationAnimaliaHemipteraIssidae

Distant, 1906

B0835CCA-B4F8-54AA-93F7-012872F9A648

Figs 29–35 , 36–43 , 44–55

Sivaloka Distant 1906: 352; Gnezdilov 2013: 42.

#### Type species.

*Sivaloka limacodes* Distant, 1906.

#### Diagnostic characters.

Body size small (Figs 30, 45). Width of head (Figs 31, 46) including eyes, narrower or broader than pronotum. Vertex (Figs 31, 46) irregularly quadrangular, with width at base more than 2.5 times longer than length at middle; disc of vertex distinctly depressed, with feeble median carina; anterior margin convexly arched; posterior margin obviously concavely arched; lateral margins parallel. Gena (Figs 32, 47) with one obvious ocellus between compound eye and antenna in lateral view. Frons (Figs 33, 48) irregularly hexagonal, with obvious median carina on basal half, feeble carina or no carina on apical half, nearly reaching to frontoclypeal suture, without lateral carinae; broader than length in middle, the base slightly narrowed, broader toward to apical margin, obviously enlarged above clypeus; lateral margins not parallel, with verrucae near lateral margins. Clypeus (Figs 33, 48) triangular, with or without median carina. Rostrum (Fig. 33) surpassing mesotrochanters. Pronotum (Figs 31, 46) triangular, with median carina or degraded, with lateral carinae; with pit each other between median carina and lateral carinae, without sub-lateral carinae; apical margin obtuse-angle convex; posterior margin nearly straight. Mesonotum (Figs 31, 46) triangular, with or without median carina, with raised sub-lateral carina. Forewings (Figs 36, 49) irregularly quadrangular, length ca 1.4–2.0 times longer than maximum width; anterior margin clearly arched; posterior margin slightly wavy; apical margin obliquely truncated in lateral view; longitudinal veins obvious, the base narrow, broader toward to the apical part; with broad “hypocostal plate”, with spherical expansion near ScP vein in basal 1/3 of forewings, ScP and RP convergent near base, ScP and RP veins long, not forked, reaching apical margin of forewing; MP bifurcating two branches before middle, MP1 forked near apical margin, MP2 forked or not near apical margin; CuA forked into brances near apical part; CuP present; Pcu and A1 united near middle of clavus, keel-shaped, especially A1 obviously keeled in lateral view. Hindwings (Fig. 37) with three lobes: with ScP-R-MP-Cu lobe developed; Pcu-A1 lobe distinctly less than half wide as ScP-R-MP-Cu lobe; A2 lobe thin, distinctly surpassing half length of Pcu-A1 lobe, anterior and posterior margins subparallel; Pcu simple, anastomosing with A11, A11 vein simple, unbranched, A12 straight and simple, A2 vein, unbranched, not reaching to apical margin. Hind tibia with 2 lateral spines in distal half and 8–10 apical spines; first metatarsomere with 8–10 apical spines; second metatarsomere with 2 apical spines; spinal formula of hind leg (2)8–10/8–10/2.

**Figures 29–35.**
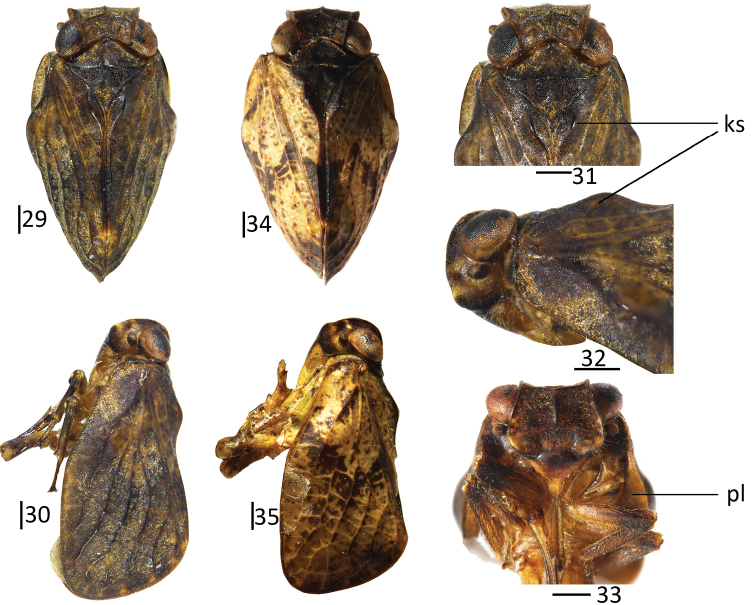
*Sivaloka arcuata* Chang & Chen, sp. nov. **29–33** holotype **34–35** paratype **29, 34** habitus, dorsal view **30, 35** habitus, lateral view **31** head and thorax, dorsal view **32** head and thorax, lateral view **33** head and thorax, ventral view. Scale bars: 0.5 mm. Abbreviations: ks = Pcu and A_1_ keel-shaped; pl = hypocostal plate.

#### Male genitalia.

Anal tube (Figs 39, 51) irregularly rectangular, elongate, longer in middle more 2.5 times than the base in dorsal view; lateral margin nearly parallel. Anal style (Figs 39, 51) long or short, not surpassing anal tube, located near base or middle. Pygofer (Figs 38, 50) symmetrical, irregularly rectangular; anterior and posterior margins parallel in lateral view. Genital styles (Figs 40, 52) symmetrical, irregularly triangular in lateral view; dorsal margin bearing different prominence before the capitulum. Capitulum of genital styles long or short (Figs 41, 53). Phallobase (Figs 42, 54) symmetrical, “U”-liked tubular in lateral view, dorsal lobe with processes near apex. Aedeagus (Figs 43, 55) with one hooked process in lateral view.

**Figures 36–43.**
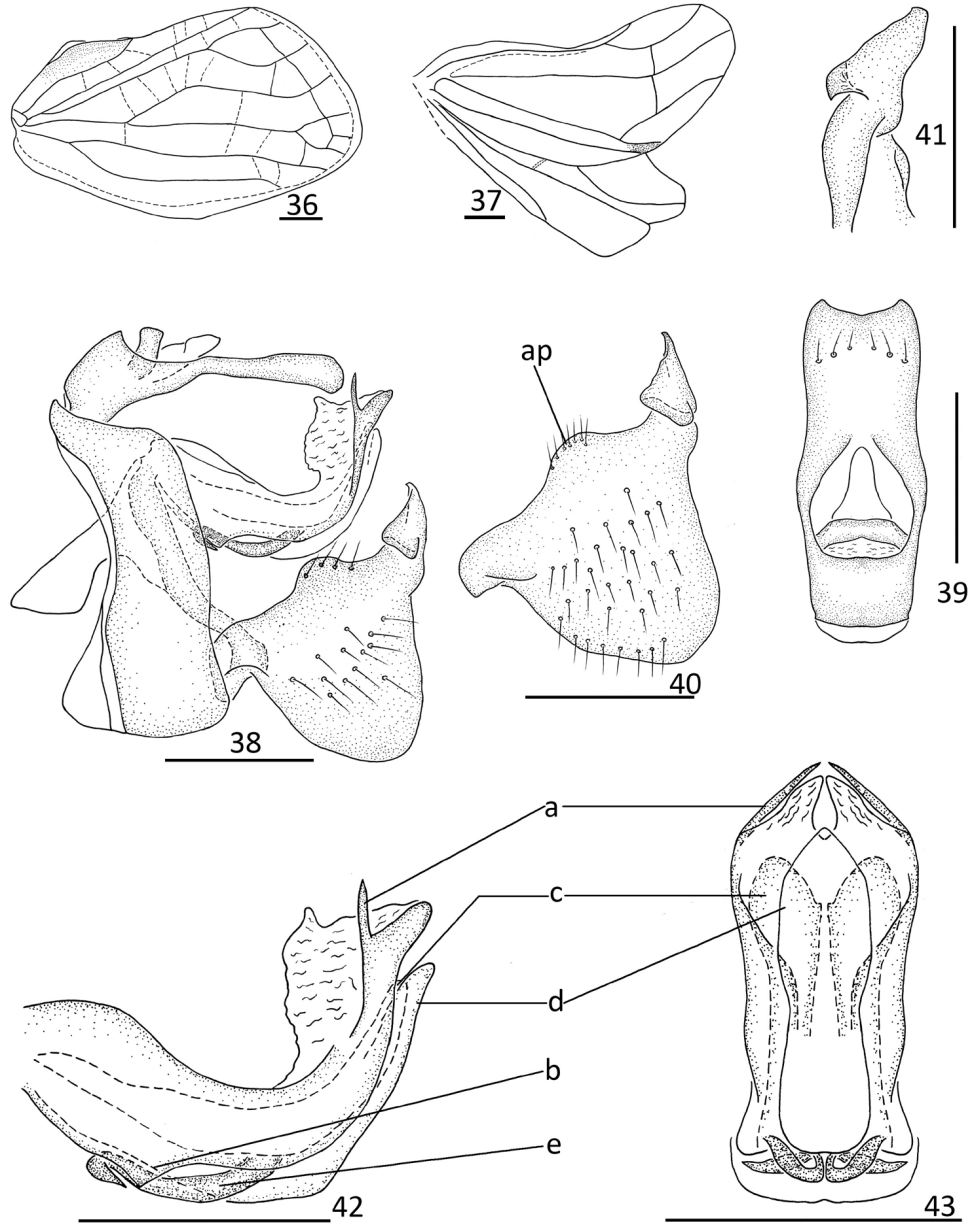
*Sivaloka arcuata* Chang & Chen, **sp. nov. 36** forewing **37** hindwing **38** male genitalia, lateral view **39** male anal segment, dorsal view **40** genital styles, lateral view **41** capitulum of genital styles, ventral view **42** phallobase and aedeagus, lateral view **43** phallobase and aedeagus, ventral view. Scale bars: 0.5 mm. Abbreviations: ap = arched prominence; a = long hooked process; b = angular process; c = lateral lobe, d = ventral lobe; e = hooked process.

#### Female genitalia.

Anal tube long, lateral margins nearly parallel. Gonoplacs irregularly rounded, without keels. Hind margin of sternite VII with prominence in middle area in ventral view.

### Key to species of *Sivaloka* Distant, 1906 of the world

**Table:** 

1	Frons with pale transverse line near middle; clypeus relatively flat, with stout median carina (Gnezdilov 2013: fig. 2)	***S. limacodes***
–	Frons without pale transverse line near middle; clypeus with basal part swollen, apical part sunken, without median carina	**2**
2	Phallobase with dorsal lobe with long hooked process near apical part (Fig. 42a); aedeagus with one long hooked process near middle in lateral view, directed to caudad (Fig. 42e)	***S. arcuata* Chang & Chen, sp. nov.**
–	Phallobase with dorsal lobe with small spinous process near apical part (Fig. 54a); aedeagus with one hooked process near basal 1/3 in lateral view, directed to cephalad (Fig. 54e)	***S. trigona* Chang & Chen, sp. nov.**

### Sivaloka arcuata

Taxon classificationAnimaliaHemipteraIssidae

Chang & Chen sp. nov.

DBDECD66-8478-57E2-94DF-235466E4FFCE

http://zoobank.org/E0F6777D-AE12-44D8-8F38-7DFCF922F2AD

Figs 29–35 , 36–43

#### Type material.

Holotype: ♂, China: Guizhou, Anlong County, Xianheping Provincial Nature Reserve (22°59'N, 105°43'E), 28 Aug. 2012, W-B Zheng leg.; paratypes: ♂, same data as holotype (IEGU); ♂, Guizhou, Congjiang County, Moon Hill (Height 1159 m) (25°38'N, 108°13'E), 19 July 2006, Q-Z Song leg.

#### Diagnosis.

This species is identified by the dark-brown or yellow-brown frons, without any bands (Fig. 33); the clypeus without median carina, with its basal part swollen and its apical part sunken (Fig. 33); the forewings longer 2.0 times than their width (Figs 30, 35); the dorsal margin of the genital styles bearing one arched prominence near the middle (Fig. 40); the phallobase with the dorsal lobe bearing a relatively long, hooked process near its apical part (Fig. 42a) and with a triangular process on the ventral margin (Fig. 42b); and the aedeagus near its middle in lateral view with one hooked process, which is directed to caudad (Fig. 42e).

#### Description.

Body length (from apex of vertex to tip of forewings): male 5.40–5.50 mm; forewings: male 4.40–4.50 mm.

#### Coloration.

General color yellow-brown or pale yellow (Figs 29, 30, 34, 35). Vertex, pronotum, and mesonotum (Fig. 31) dark-brown, suffused with rusty brown. Gena (Fig. 32) dark brown, with two inconspicuous yellow bands. Compound eyes brown to black, ocelli yellow (Fig. 32). Frons (Fig. 33) dark brown or yellow-brown, with yellow verrucae near lateral margin. Clypeus (Fig. 33) with basal part black, apical part yellow to yellow-brown. Forewings (Figs 29, 34) dark brown or pale yellow, with diffuse rusty brown. Hindwings brown. Legs yellow-brown, tip of spineson hind tibiae and tarsi black.

#### Head and thorax.

Head (Fig. 31) including eyes, slightly narrower than pronotum (0.93: 1.00). Vertex (Fig. 31) shorter in middle than width at base (0.50: 1.00). Frons (Fig. 33) shorter in middle than maximum breadth (0.65: 1.00); with median carina distinct, reaching to the level of middle of frons. Clypeus (Fig. 33) triangular, without median carina; basal part swollen, apical part slightly sunken (Fig. 33). Pronotum (Fig. 31) with median carina feeble. Mesonotum (Fig. 31) with median carina raised, fused in anterior margin. Forewings (Fig. 36) longer than wide (2.00: 1.00); with broad “hypocostal plate”, ScP and RP convergent near base, short common stem, ScP and RP veins long, parallel with anterior margin of forewing, reaching to apical margin; MP two branched in basal 1/3, MP1 dividing two branches in distal 1/3, MP2 not forked in distal part; CuA forked into two branches in distal 1/3, CuP present; Pcu and A1 united in middle of clavus, clavus almost reaching to 2/3 of forewing. Hindwings (Fig. 37) with ScP+R and CuA forked near apical part, MP simple, not forked, CuA2 and CuP fused near apical part, with one vein between R and M, M and CuA1; Pcu and A11 unbranched, with one transverse vein between Pcu+A11 and A12; A2 reaching to apical 1/3 of A2 lobe. Spinal formula of hind leg (2)8–10/8–10/2.

#### Male genitalia.

Anal tube in dorsal view (Fig. 39) longer in middle than the widest breadth (2.50: 1.00), the maximum width in middle of anal tube; apical margin distinctly, angularly concave; lateral margins almost parallel, slightly concave near apical 2/3 of anal tube. Anal style (Fig. 39) relatively long and stout, located in basal 2/5 of anal tube, not surpassing the opening of anal pore. Pygofer (Fig. 38) irregularly rectangular; anterior and posterior margins nearly parallel in lateral view; dorsal and ventral margins parallel. Genital styles (Fig. 40) irregularly triangular in lateral view; dorsal and ventral margins not parallel; dorsal margin with one arched prominence near middle at base of capitulum; ventral margin slightly arched. Capitulum of genital styles irregularly triangular, with small lobe; with stout and not obvious neck (Fig. 41). Phallobase (Figs 42, 43) with dorsal lobe slightly expanded into membranous, cystiform process; and dorso-lateral lobe splitting into relatively long, hooked process near apical part (Figs 42a, 43a), ventral margin of dorso-lateral lobe with angular process in basal 1/3 in lateral view (Fig. 42b); lateral lobe distinctly shorter than dorsal lobe in lateral view (Fig. 42c), splitting into two stout branches (Fig. 43c); ventral lobe relatively longer than lateral lobe in lateral view (Fig. 42d) in lateral view; in ventral view, apical part obviously archedly convex (Fig. 43d); lateral margins parallel in ventral view. Aedeagus (Figs 42, 43) with one long, hooked process near middle (Fig. 42e) in lateral view, directed to caudad.

#### Etymology.

The specific name is derived from the Latin words “arcuata” in reference to the genital styles which bear an arched prominence on the base before the capitulum.

#### Host plant.

Unknown.

#### Distribution.

China (Guizhou).

#### Remarks.

The new species is similar to *S. limacodes* Distant, 1906, but it differs from it by: 1) frons dark brown or yellow-brown, without any band (Fig. 33) (frons with pale and transverse line near middle in *S. limacodes*); 2) clypeus without median carina, basal part swollen, apical part sunken (Fig. 33) (clypeus with stout median carina, relatively flat in *S. limacodes*) ; 3) forewings 2.00 times longer than their maximum breadth (Fig. 36) (forewings 1.40 times longer than their maximum breadth in *S. limacodes*).

### Sivaloka trigona

Taxon classificationAnimaliaHemipteraIssidae

Chang & Chen sp. nov.

803A80FF-6D64-5E08-80FE-B42C978DC531

http://zoobank.org/D273CBAC-17A5-4B0B-9E6B-850C4ABE9386

Figs 44–55

#### Type material.

Holotype: ♂, China: Guangxi, Yangshuo County (24°59'N, 105°36'E), 28 May 2009, W-B Zheng leg. (IEGU).

#### Diagnosis.

This species is similar to *S. arcuata* Chang & Chen, sp. nov., but it differs from the latter by: 1) forewings with MP_2_ dividing into two branches (Fig. 49); 2) dorsal margin of genital styles bearing one triangular prominence near middle (Fig. 52); 3) phallobase with dorsal lobe with a small spinous process near apical part (Fig. 54a), ventral margin with half-leaf process in basal 1/3 (Fig. 54b); 4) aedeagus in lateral view with one short, hooked process near basal 1/3, directing to cephalad (Fig. 54e).

#### Description.

Body length (from apex of vertex to tip of forewings): male 5.00 mm; forewings: male 4.10 mm.

#### Coloration.

**Figures 44–55.**
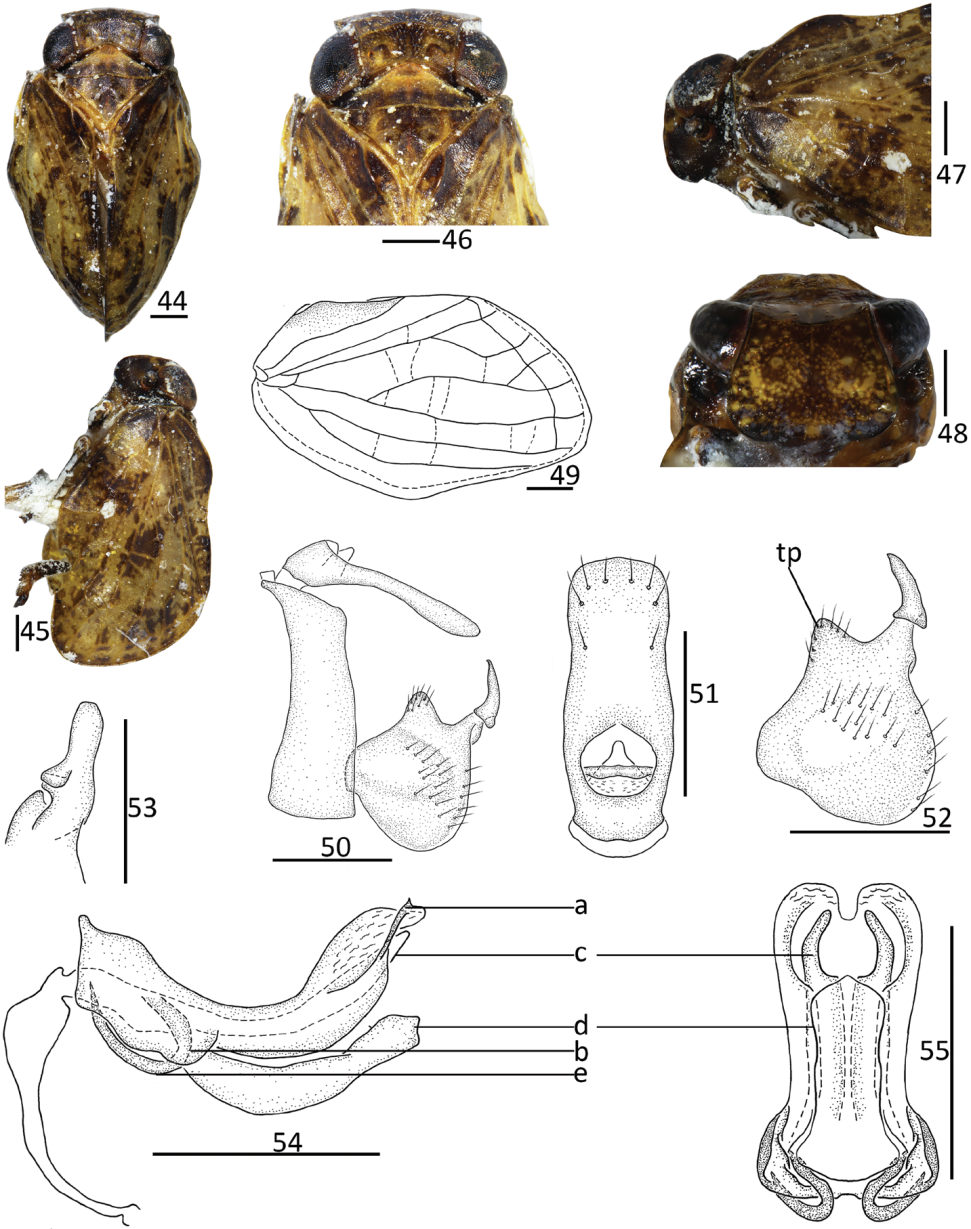
*Sivaloka trigona* Chang & Chen, sp. nov. **44** habitus, dorsal view **45** habitus, lateral view **46** head and thorax, dorsal view **47** head and thorax, lateral view **48** head, ventral view **49** forewing **50** male genitalia, lateral view **51** male anal segment, dorsal view **52** genital styles, lateral view **53** capitulum of genital styles, ventral view **54** phallobase and aedeagus, lateral view **55** phallobase and aedeagus, ventral view. Scale bars: 0.5 mm. Abbreviations: tp = triangular prominence; a = small spinous process; b = half-leaf process; c = lateral lobe; d = ventral lobe; e = hooked process.

General color pale yellow (Figs 44, 45). Vertex, pronotum, and mesonotum (Fig. 46) pale yellow to brown. Gena (Fig. 47) dark brown, with one not obvious yellow band. Compound eyes and antennae black, ocelli pale (Fig. 47). Frons (Fig. 48) dark brown, with scores of pale verrucae along lateral margin. Clypeus black-brown (Fig. 48). Forewings (Figs 44, 45) pale yellow, with diffuse, dark brownish markings. Hindwings brown. Legs pale green or yellow-brown, tip of spines on hind tibiae and tarsi black.

#### Head and thorax.

Head (Fig. 46) including eyes, slightly broader than pronotum (1.07: 1.00). Vertex (Fig. 46) shorter in middle than the width at base (0.41: 1.00). Frons (Fig. 48) slightly shorter in middle than maximum breadth (0.69: 1.00); with median carina, its basal half distinct, reaching to the level of middle of frons, apical part feeble, nearly to the frontoclypeal suture. Clypeus (Fig. 48) triangular, with median carina; basal part swollen, apical part slightly sunken. Pronotum (Fig. 46) with median carina feeble. Mesonotum (Fig. 46) with median carina raised, basal part forked, fused in anterior margin; lateral carinae not obvious. Forewings (Fig. 49) 1.63 times as long as maximum breadth; ScP and RP convergent near base, ScP and RP long, parallel to anterior margin of forewing, reaching to outer margin; MP two branches in basal 1/3, MP_1_ in distal 1/5 dividing into two branches or unbranched, MP_2_ forked in distal 1/5; CuA forked into two branches in distal 1/3; CuP present; Pcu and A_1_ united in middle of clavus, clavus almost reaching to 2/3 of forewing. Hindwings unknown. Spinal formula of hind leg (2)9/9/2.

#### Male genitalia.

Anal tube in dorsal view (Fig. 51) longer in middle than at widest breadth (2.57: 1.00), maximum width at middle of anal tube; anterior margin almost straight; lateral margins parallel, lateral margin slightly concave near apical 2/5. Anal style (Fig. 51) short and thin, located in basal 2/5, not surpassing the opening of anal pore. Pygofer (Fig. 50) irregularly rectangular; anterior and posterior margins parallel in lateral view; dorsal margin inclined to ventral margin. Genital styles (Fig. 52) irregularly triangular in lateral view; dorsal and ventral margins not parallel; dorsal margin with triangular prominence near middle at base of capitulum; ventral margin slightly arched. Capitulum of genital styles irregularly triangular; with irregular triangular, and thin, distinct neck (Fig. 53). Phallobase (Figs 54, 55) with dorsal lobe slightly expanded into membranous cystiform process; dorso-lateral lobe with a small spinous process near apical part (Fig. 54a) in lateral view; ventral margin with half-leaf process in basal 1/3, margin wavy (Fig. 54b); lateral lobe shorter than dorsal lobe (Fig. 54c), splitting into two branches, apical part appearing long thin finger, and basal part stout in ventral view (Fig. 55c); ventral lobe obviously shorter than lateral lobe in lateral view (Fig. 54d); in ventral view, apical margin of ventral lobe subtriangular and convex in middle; lateral margin of ventral lobe parallel in ventral view (Fig. 55d). Aedeagus (Figs 54, 55) with one relatively short, hooked process near basal 1/3 (Fig. 54e) in lateral view, directed to cephalad.

#### Etymology.

The specific name is derived from the Latin words “*trigona*” in reference to the triangular prominence near the middle of the dorsal margin of the genital styles.

#### Host plant.

Unknown.

#### Distribution.

China (Guangxi).

#### Remarks.

The new species is similar to *S. arcuata* Chang & Chen, sp. nov. in appearance, but it differs the latter by: 1) dorsal margin of genital styles bearing one triangular prominence near middle (Fig. 52) (dorsal margin of genital styles bearing one arched prominence near middle in *S. arcuata* (Fig. 40)); 2) phallobase (Fig. 54) with dorsal lobe with a small spinous process near apical part (Fig. 54a), ventral margin with half-leaf process in basal 1/3 (Fig. 54b) (phallobase with dorsal lobe with long hooked process near apical part in *S. arcuata* (Fig. 42a), ventral margin with triangular process (Fig. 42b)); 3) aedeagus (Fig. 54) with one hooked process near basal 1/3 in lateral view, directed to cephalad (Fig. 54e)(aedeagus with one hooked process near middle in lateral view, directed to caudad (Fig. 42e))

## Discussion

A comparison of *Kodaianella* Fennah, 1956, *Sivaloka* Distant, 1906, and *Dentatissus* Chen, Zhang & Chang, 2014, shows that species in these genera look rather similar. In these genera the width of the vertex at the base is longer than its length at its middle, the frons lacks transverse carina, the hingwings have A_11_ unbranched, and the male genitalia are in general similar.

*Sivaloka* is, however, clearly different from the other two genera in having the Pcu and A_1_ veins on the forewings keel-shaped (Fig. 2; Chen et al. 2014: fig. 2–79B). There are also significant generic differences in the structure of the male genitalia among the three genera. In *Sivaloka*, the anal tube is irregularly rectangular with its lateral margins nearly parallel (Figs 39, 51), while in *Kodaianella* the anal tube is irregularly triangular with the lateral margin apically diverging (Fig. 9); in *Dentatissus* the anal tube is oval and wider near its middle (Chen et al. 2014: fig. 2–79H). The capitulum of the genital styles are with a stout irregular triangular at their base in *Dentatissus* (Chen et al. 2014: fig. 2–79J); the capitulum are absent *Kodaianella* and *Sivaloka* (Figs 10, 40). The dorsal lobe of the phallobase bears various processes near its apical part in *Kodaianella* and *Sivaloka* (Figs 12, 42), but these processes are absent in *Dentatissus*. The aedeagus bears two pairs of hook-like processes in *Dentatissus* (Chen et al. 2014: fig. 2–79J) and one pair of hook-like processes in *Kodaianella* and *Sivaloka*.

## Supplementary Material

XML Treatment for Kodaianella

XML Treatment for Kodaianella furcata

XML Treatment for Kodaianella bicinctifrons

XML Treatment for Kodaianella longispina

XML Treatment for Sivaloka

XML Treatment for Sivaloka arcuata

XML Treatment for Sivaloka trigona
